# Ireland's silent epidemic: Why FASD can't be ignored anymore

**DOI:** 10.3389/fpubh.2026.1781199

**Published:** 2026-04-28

**Authors:** Angel Harper, Farhana Sharif, Tristan Casson-Rennie, Jolanta Burke

**Affiliations:** 1 Royal College of Surgeons in Ireland (RCSI), Centre for Positive Health Sciences, Dublin, Ireland; 2 Paediatrics Unit, Mullingar Regional Hospital, Mullingar, Ireland; 3 Department of Paeditrics, University College Dublin, Dublin, Ireland; 4 FASD Ireland, Ennis, Ireland

**Keywords:** FASD, Foetal Alcohol Spectrum Disorder, health and wellbeing, Irish healthcare, neurodevelopmental disorder

## 1 Introduction

Historically, pregnant women in Ireland were advised to drink stout for its supposed iron-boosting benefits (1), a myth which has become culturally ingrained across parents and medical professionals. Many parents or parents-to-be have reported a mixed understanding of safe and responsible drinking practices when trying for a baby, with one participant even recalling their midwife telling them that “a glass of red wine was actually better for the baby” (2). Such misconceptions can lead to dangerous drinking by parents leading up to and during pregnancy, resulting in Foetal Alcohol Spectrum Disorder (FASD), the most under-recognized neurodevelopmental condition worldwide. FASD continues to affect millions of individuals and families, who are often left unsupported and stigmatized. FASD impacts people universally, causing stress to families (3), mental health challenges for individuals (4), and increased financial burdens on education, healthcare, criminal justice, and productivity (5). Despite this, there are few resources for diagnosis or support. Ireland in particular, has one of the highest prevalence rates globally (6), and yet still does not recognize FASD as a disability (7).

### 1.1 What is FASD?

FASD is an umbrella term, encompassing a wide range of severe and complex lifelong neurodevelopmental, physical, and behavioral conditions caused by prenatal alcohol exposure. Drinking by either parent leading up to conception or during pregnancy increases the risk of FASD. Symptoms vary widely but can include growth deficiencies, hearing loss, cognitive difficulties, learning disabilities, and issues with memory, attention, and emotional regulation (8). Importantly, FASD is not always visible. Only 10% of individuals display the facial characteristics associated with Foetal Alcohol Syndrome (9): small eye openings, thin upper lip, and a smooth groove between the nose and lip. Consequently, FASD is often overlooked and frequently misdiagnosed as autism or ADHD (10). For example, impulsivity and attention difficulties are present in both FASD and ADHD, with 63% of those with FASD also meeting the criteria for ADHD (11). A study of 156 children found an 80.1% missed diagnosis rate (12), leaving many without appropriate support or treatment.

### 1.2 The impact of FASD

Across the world, FASD impacts millions of people; a meta-analysis investigating the prevalence of FASD globally found that for every 1,000 members of the general population, eight are living with FASD (6). In fact, the prevalence rates are higher than 1% in 76 countries, with the highest rates found in South Africa, Croatia, Ireland, Italy, and Belarus. These findings highlight the need for public health policy changes on a global scale.

FASD impacts children from birth, and without appropriate diagnosis and support, can result in adverse life outcomes such as school expulsion, alcohol and drug problems, and challenges with law enforcement agencies; with the age of diagnosis associated with increased likelihood of these outcomes occurring. In fact, the chances of adverse life outcomes occurring increase by 2–4 times if they received a diagnosis after the age of 12 (13). Mental illness is also prevalent, with leading causes of death in those living with FASD being by suicide, accidents, and drug or alcohol abuse (14). This vulnerable population are at greater risk of adverse life outcomes, especially in the absence of appropriate diagnosis and a framework of support, highlighting the urgent need for improved FASD services.

FASD also impacts caregivers, especially birth mothers, who face significant stigma from the community. Focus groups and interviews revealed that respondents perceived biological mothers as abusive or negligent (15). Such stigma can prevent parents from coming forward and therefore receiving the support they or their child needs. In addition, it ignores the role that men's drinking plays in the development of FASD, as fathers-to-be should also refrain from drinking leading up to and during conception. Ultimately, shame and judgement do not prevent FASD and certainly do not help those living with it.

### 1.3 FASD in Ireland

Ireland is estimated to have the third highest prevalence of FASD worldwide, with as many as 7.4% of the population potentially living with FASD (6). Based on this estimate, someone with 150 acquaintances would likely know 11 people living with FASD, a teacher with 30 students would know 2, a doctor with 15 patients a day would have met at least 1. Despite this, Ireland has no official diagnostic standard or pathway to diagnosis for FASD.

In a survey of Irish carers, many reported the inadequacies of the current healthcare system for FASD diagnosis and support (16). 73% of carers had been able to confirm a diagnosis for their child, but 40% stated that their child had not received needed medical care due to a lack of services available. The majority (89%) also felt that FASD services were “somewhat or extremely inadequate.” Half of respondents reported their children had no adapted education plans, with many feeling misunderstood by schools. Carers also reported significant financial and emotional strain throughout the diagnosis and caregiving journey; parents of those living with FASD have reported the emotional toll their dissatisfaction with the current healthcare system in Ireland.

A recent report which interviewed Irish carers and people living with FASD described how many felt neglected by the education and healthcare sectors (17). They faced challenges within the Irish healthcare system, and many were unable to receive a formal diagnosis for their child, stating that they “are blamed but they're not supported.” People living with FASD often felt excluded by peers, many reported incidents of bullying or being taken advantage of as a result of their vulnerability. In addition, this report conducted a poll which found that 82% had never heard of FASD. This lack of support and FASD awareness not only prevents diagnosis but also leaves families navigating FASD on their own.

Currently, Ireland offers no official diagnostic standard, no pathways for diagnosis, no framework of support, and no recognition of FASD as a disability. Previous research indicates that not receiving a diagnosis from an early age can increase the likelihood of adverse life outcomes, and that this population are at greater risk of mental illness and substance abuse (13, 14). A national HSE funded FASD clinical practice and a coordinated public health approach are urgently needed to protect the FASD community, and support prevention efforts.

## 2 Discussion: What needs to be done

Ireland is facing a public health crisis; despite having one of the highest estimated rates of FASD in the world, the Irish system is ill-equipped to diagnose, support and recognize this condition. The existing international research shows unequivocally that early diagnosis and intervention are essential for young people living with FASD to help them live a fulfilling life. Yet, Ireland continues to lag behind, leaving children and their families alone to navigate through this complex disability.

The recommendations highlighted in a recent report on FASD in Ireland (17) make it clear that urgent change is required. Firstly, the Irish government and HSE need to formally recognize FASD as a disability. Until then, those living with FASD will remain invisible within the systems which are meant to protect them. The United Nations on the Rights of Persons with Disabilities (18) is an international treaty dedicated to protecting the rights of disabled people. As a member, Ireland has both ethical and legal obligations to acknowledge FASD, protect the rights of those living with it, offer targeted supports, and introduce inclusive FASD policies. Anything else is a dereliction of duty of care.

However, recognition alone is not enough to make a change. Public awareness and education are required to prevent, diagnose, and support. Nationwide education must become a priority teaching the public about the risks of either parents' alcohol consumption leading up to and throughout pregnancy. Other countries have shown that public campaigns work, so Ireland has no excuse not to follow suit. This could involve TV advertisements, radio announcements, clearer labeling on alcoholic products, pamphlets at medical centers, or townhall educational events. Previous campaigns carried out in other countries have shown success in relaying campaign messages (19, 20). Furthermore, FASD training should be a mandatory requirement for teachers, special needs assistants, doctors, pediatricians, social workers, or anyone working with children; parents-to-be of all genders need to be informed of the risks of drinking when planning to start or grow their family and the characteristics of FASD in a non-judgemental way. Medical professionals should also complete training for a diagnostic tool, the four-digit diagnostic code (21), which is freely available online. Without this educational infrastructure, prevention and early intervention will remain unattainable.

Equally urgent is the need to establish a national HSE-funded FASD clinic with an appointed FASD clinical lead. Caregivers often report long waiting lists, inadequate treatment approaches and a lack of diagnosis options, leaving families feeling exhausted and unsupported (16). Many reach out to healthcare systems outside of Ireland, such as Canada. These gaps in support not only frustrate them, but may also cause harm to the young people affected, who are desperately waiting for treatment (13). Delayed diagnosis increases the risk of poor mental health outcomes and may lead to lifelong challenges, which could be mitigated with early intervention. A streamlined diagnosis is not only a recommendation but a necessity.

Childhood diagnosis and early intervention are essential, but they cannot remain confined to childhood. People living with FASD require life-long support. Currently, adult FASD services in Ireland are almost non-existent. Regional clinics or community groups which can offer advice, mental health support, and career services for adults could aid independence and reduce the risk of mental illness and substance abuse. Research suggests receiving a diagnosis in adulthood improves access to necessary supports/ resources, resulting in greater understanding of themselves and adaptation into adult life (22). Furthermore, adults living with FASD who received mentorship reported increased quality of life (23). FASD is lifelong, so the supports offered in Ireland should be too.

Environmental accommodations need to be put in place to support both children and adults living with FASD. Schools can offer adapted learning plans, which can tailor support for hyperactivity, emotional regulation, or learning difficulties. Access to emotional regulation tools such as movement breaks, fidget toys, or the Zones of Regulation are just a few examples of how schools can adapt their environment for children living with FASD. For adults, workplaces may need to introduce an FASD-friendly environment, such as using dyslexic friendly fonts in emails, allowing for a flexible work schedule, or explaining instructions slowly and clearly. Creating an inclusive environment allows individuals living with FASD greater quality of life, fostering inclusivity and empathy within institutions.

Perhaps one of the most considerable gaps that exists is Ireland's lack of research. Whilst research has estimated Ireland has the third highest prevalence, Ireland itself has not conducted its own prevalence research on FASD. This is a huge oversight, as more accurate estimates, particularly across regions and specific demographics, can offer targeted prevention and support for high-risk groups. For instance, in the UK, knowledge of alcohol guidelines varies across gender, age, and region (24), indicating demographic differences in knowledge. Additionally, there is little knowledge of how FASD impacts the health and wellbeing of Irish citizens, the knowledge of the condition in the general public, and the financial strain FASD places on various sectors including education, healthcare, and the criminal justice system. Without adequate data or prevalence across regions, demographic differences in alcohol consumption knowledge, or the economic cost of FASD, Ireland is legislating in the dark. It is of utmost importance to change this.

Finally, change is also required at individual and cultural levels. Ireland's relationship with alcohol is deeply ingrained in the society and rarely questioned. Yet, alcohol consumption by either parent in the 64 days prior to conception (25), and by mothers throughout pregnancy can impact a child's lifelong development. The normalization of drinking within Irish culture can be challenged through engaging in respectful conversations with friends and family members or organizing alcohol-free social events. Additionally, approaching others with kindness and understanding, rather than judgement, can facilitate safer attitudes toward coming forward for a diagnosis, or with concerns about substance abuse. Individual change can therefore work alongside systematic policy reform, creating a more educated, supportive, and resilient community.

In conclusion, Ireland faces a silent epidemic. Despite one of the highest prevalence rates in the world, FASD services are nearly non-existent. This could have devastating consequences for those living with FASD and their families. Education and policy change is required to improve FASD services for the estimated 381,000 people living with FASD in Ireland today. This Irish context may therefore serve as an important case study for other countries seeking to address the persistent global gap between FASD prevalence and the availability of appropriate diagnostic and support services. People living with FASD have a strong self-awareness, capacity for human connection, resilience, and hope for the future (26)–let's honor this hope by ensuring that, as a society, we can truly say: we see you.
